# A comprehensive model with dual plane ultrasound based deep learning radiomics to distinguish parotid pleomorphic adenoma from Warthin tumor

**DOI:** 10.3389/fonc.2026.1890152

**Published:** 2026-07-24

**Authors:** Yi Wang, An Dai, Zhaolin Yin, Jiening Gao, Meng Sun, Yufei Xiao, Jingyu Chen, Ruoling Han

**Affiliations:** 1 Department of Ultrasound, The Fourth Hospital of Hebei Medical University, Shijiazhuang, Hebei, China; 2 Department of Ultrasound, Tangshan Gongren Hospital, Tangshan, Hebei, China

**Keywords:** deep learning, dual-plane, pleomorphic adenoma, radiomics, ultrasound, Warthin tumor

## Abstract

**Objective:**

To develop and validate a dual-plane ultrasound (US)-based deep learning radiomics model for the noninvasive discrimination between parotid pleomorphic adenoma (PA) and Warthin tumor (WT).

**Methods:**

This retrospective two-center study enrolled 656 patients with pathologically confirmed parotid tumors (361 PA, 295 WT). Cases from Center 1 (n = 556) were randomly split into training and internal validation sets (7:3), while Center 2 cases (n = 100) formed the external test set. Radiomics features were extracted from long-axis and short-axis plane US images respectively to construct dual-plane radiomics models. For deep learning, pre-trained ResNet/DenseNet was adopted, and two fusion strategies were compared: multi-channel image fusion (long-axis, short-axis, and their pixel-wise mean) versus feature fusion (concatenation of deep features extracted from each plane). A comprehensive prediction model (nomogram) was further constructed by integrating clinical information and US features. Performance was evaluated using AUC, calibration curves, and DCA, and compared against three US physicians of varying experience. Diagnostic performance with and without model assistance was also assessed.

**Results:**

The multi-channel fusion deep learning model (AUC = 0.860, 95%CI: 0.790-0.931) outperformed feature fusion (AUC = 0.818, 95%CI: 0.735-0.904). Nomogram achieved the highest AUC: 0.907(95%CI: 0.850-0.964) in testing set, outperforming individual clinical, radiomics and deep learning models. It also exceeded the diagnostic performance of all physicians. Model assistance improved junior physicians’ AUC by 0.062 and specificity by 0.220; senior and expert physicians showed higher sensitivity and accuracy.

**Conclusions:**

The proposed dual-plane US-based deep learning radiomics model effectively differentiates PA from WT noninvasively, enhances diagnostic performance—particularly for less experienced physicians—and demonstrates potential for clinical translation.

## 1 Introduction

Parotid gland tumors exhibit diverse histopathological types, with approximately 75%-80% being benign. The most common types are pleomorphic adenoma (PA) and Warthin tumor (WT) (1, 2). PA accounts for about 60%-70% of benign parotid tumors and occurs predominantly in women aged 40–60 years (3, 4). Compared to other benign parotid tumors, PA has a higher tendency for recurrence and malignant transformation. Surgical resection is the primary treatment, often involving partial or total superficial parotidectomy (5, 6). WT, also known as adenolymphoma or papillary cystadenoma lymphomatosum, is commonly found in male smokers aged 50–70 years. It typically presents as a painless, slow-growing solid mass with a low recurrence rate (approximately 4.2%) and minimal risk of malignant transformation (about 1%). Consequently, the current treatment trend favors less extensive surgical approaches, such as extracapsular dissection, to reduce the risk of facial nerve injury. For elderly patients with surgical contraindications or multiple comorbidities, conservative observation may also be considered (7, 8). Therefore, accurate preoperative differentiation between PA and WT is crucial for treatment decision-making and prognostic evaluation.

Preoperative diagnosis of parotid gland tumors primarily relies on fine-needle aspiration cytology (FNAC), core needle biopsy (CNB), and imaging modalities. However, FNAC and CNB are associated with limited tissue sampling and carry potential risks of tumor seeding or iatrogenic sialadenitis (9, 10). Commonly used imaging techniques include ultrasound (US), computed tomography (CT), and magnetic resonance imaging (MRI). Among these, high-resolution US has become the preferred initial screening modality due to its advantages such as absence of ionizing radiation and real-time dynamic imaging capability (11). Two-dimensional US allows clear visualization of tumor morphology, while color doppler flow imaging provides valuable information on vascularity. Furthermore, US-guided biopsy plays a significant role in clinical practice. Nevertheless, imaging features of PA and WT often overlap, and interpretation is subject to interobserver variability and operator dependence, leading to diagnostic uncertainty (12). Therefore, there is an urgent need for reliable, non-invasive diagnostic approaches. Artificial intelligence-based image analysis offers a promising avenue to address this challenge.

Medical image analysis is a data-driven computational task. Radiomics enables high-throughput extraction of quantitative features from medical images, capturing complex spatial and statistical relationships among voxels (13). As another key branch of machine learning, deep learning (DL) automatically learns hierarchical semantic and spatial representations through multilayer neural networks, thereby comprehensively characterizing lesion phenotypes (14). Recent evidence indicates that integrated models combining radiomics and deep learning outperform single-modality approaches in various domains (15–17). In parotid gland tumor research, CT or MRI-based studies have achieved tumor detection, classification, and PA-WT differentiation (18–21). US-based studies, however, remain limited (22, 23). Furthermore, most current US studies are confined to single-plane images or single-source features. The integration of multi-source information and impact of fusion strategies on model performance warrant further investigation.

This study aims to develop and validate a comprehensive US-based model integrating clinical and US features, radiomics, and deep learning for preoperative differentiation of PA from WT. Unlike previous single-plane studies, the main contributions of this study include (1): dual-plane acquisition—simultaneously utilizing long- and short-axis images acquired based on the maximum diameter to extract richer spatial and structural information; (2) comparison of fusion strategies—systematically comparing multi-channel image fusion versus feature fusion on model performance; (3) clinical utility assessment—comparing model performance with interpretations by US physicians of varying experience levels before and after model assistance to evaluate its clinical translatability.

## 2 Materials and methods

### 2.1 Patients

**Figure 1:**
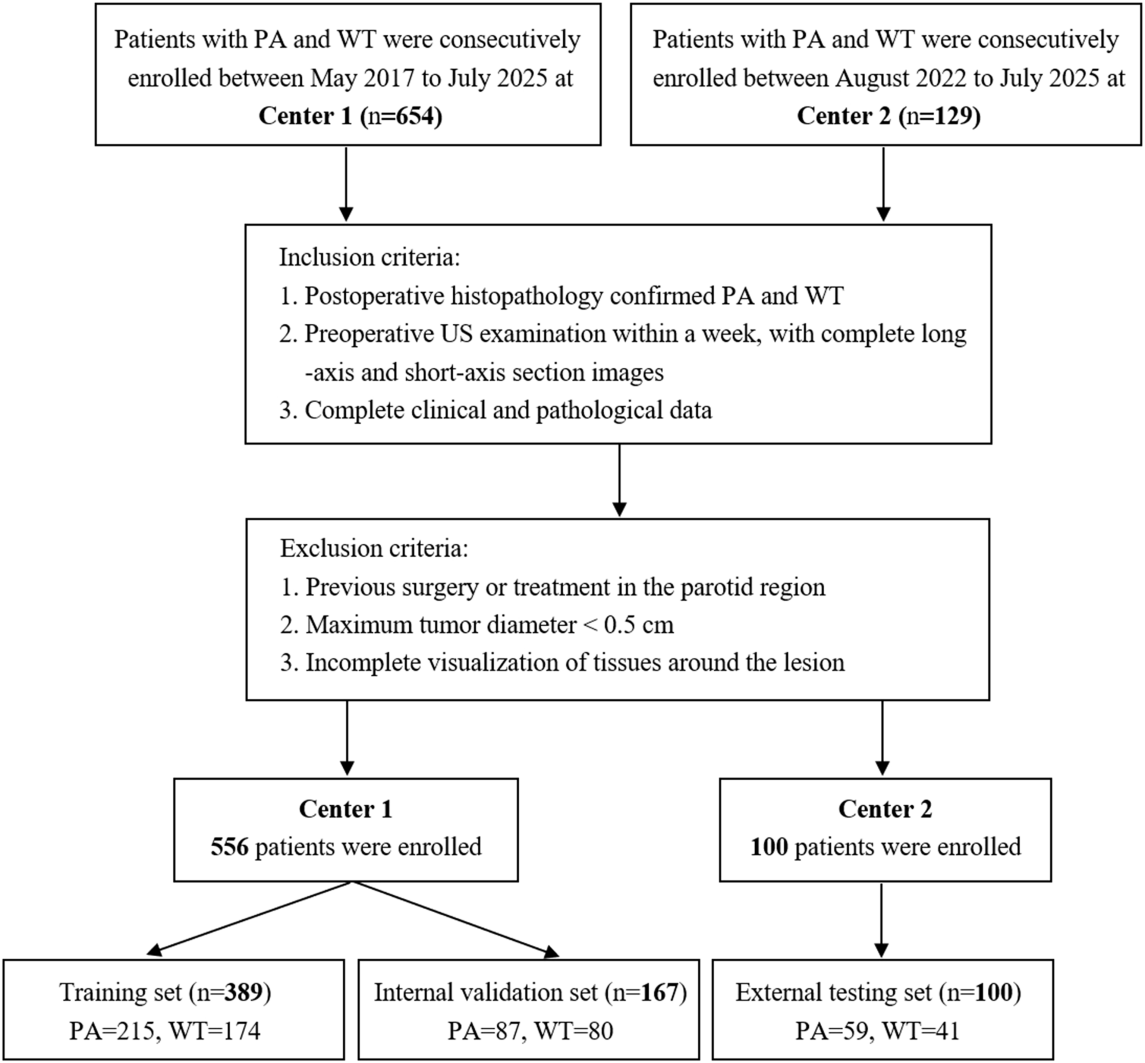
Flowchart of inclusion and exclusion for each data set. PA, pleomorphic adenoma; US, ultrasound; WT, Warthin tumor.

This retrospective study collected 783 patients with histopathologically confirmed primary PA and WT from two medical centers. Of these, 654 patients were enrolled from Center 1 (The Fourth Hospital of Hebei Medical University, May 2017 to July 2025) and 129 from Center 2 (Tangshan Gongren Hospital, August 2022 to July 2025). This study was conducted in accordance with the guiding principles of the Declaration of Helsinki and approved by the institutional ethics committees (Ethics Approval Number: 2024KS002). As a retrospective study, informed consent from patients was waived. Clinical and imaging data were retrieved from the Picture Archiving and Communication System (PACS) and the Hospital Information System (HIS). Inclusion criteria were as follows: (1) histopathological diagnosis of primary PA or WT; (2) US examination performed within one week prior to surgery, with complete long-axis and short-axis sectional images available; (3) complete clinical records, including detailed smoking and alcohol consumption histories. Exclusion criteria were: (1) history of prior parotid region surgery or treatment; (2) tumor maximum diameter< 0.5 cm; (3) poor image quality or incomplete visualization of the lesion due to large tumor size. For patients with multiple lesions, the largest or most representative lesion was selected for analysis. The patient selection workflow is illustrated in **Figure 1**.

**Figure 2:**
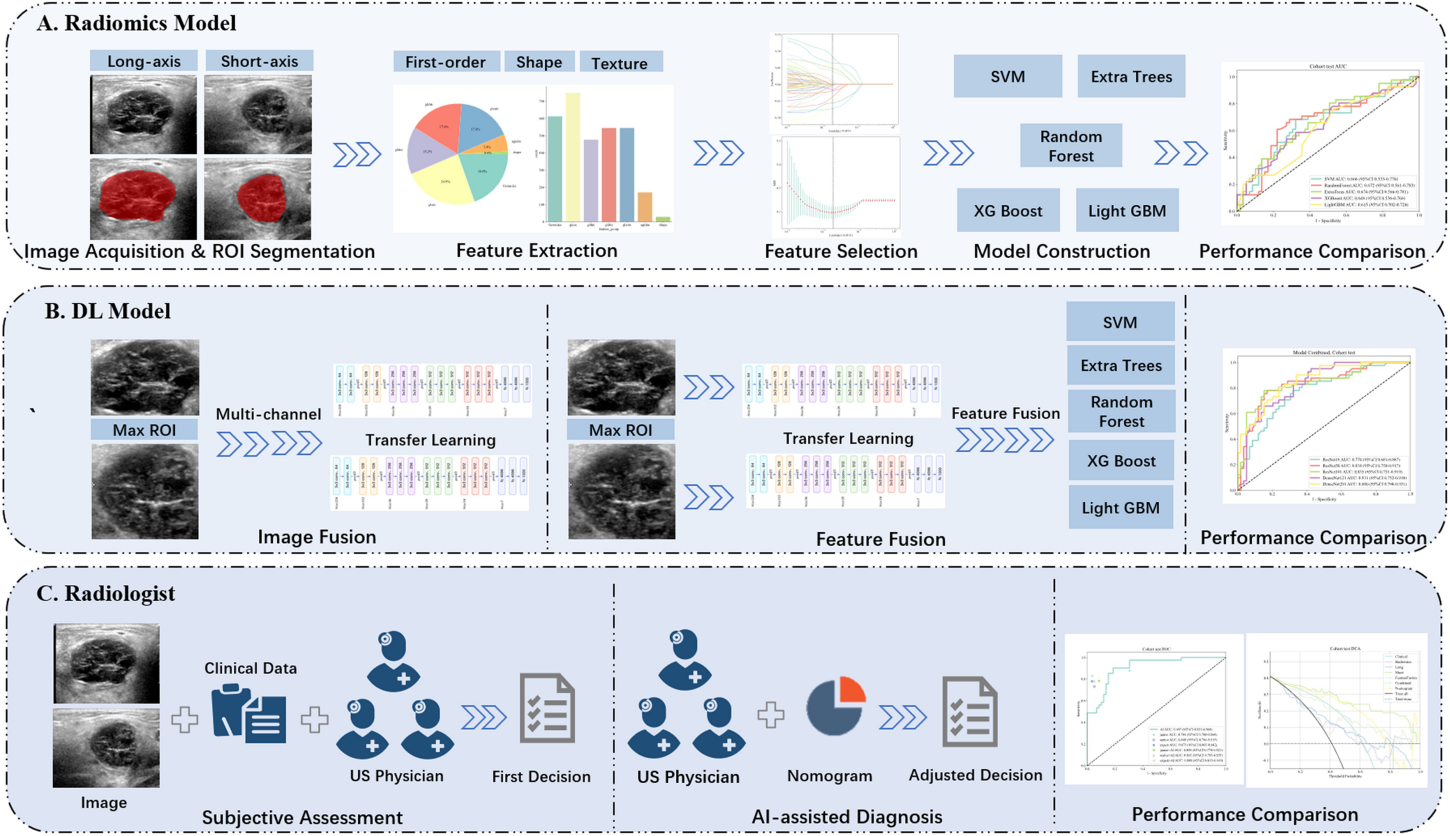
The overall workflow of this study. AI, artificial intelligence; DL, deep learning; ROI, region of interest. US, ultrasound.

A total of 656 patients were included in the final analysis, with 556 from Center 1 (302 PA and 254 WT) and 100 from Center 2 (59 PA and 41 WT). Cases from Center 1 were randomly assigned to a training set and an internal validation set at a 7:3 ratio, while data from Center 2 served as an independent external testing set. The study design and workflow are summarized in **Figure 2**.

### 2.2 Image acquisition and analysis

All patients underwent detailed preoperative examination of the parotid region using ultrasonic instruments including iU22 (PHILIPS), EPIQ7C (PHILIPS), S2000 (SIEMENS), and ACUSON Sequoia (SIEMENS), equipped with corresponding high-frequency linear array transducers. Long-axis grayscale images at the maximum tumor diameter and corresponding orthogonal short-axis images were retrieved from PACS for subsequent analysis.

US images were independently evaluated by three US physicians: a junior physician (A, 3 years of experience in superficial organ ultrasound), a senior physician (B, 13 years of experience), and an expert (C, over 20 years of experience). The following US features were recorded: maximum lesion diameter, shape (irregular/lobulated vs. round/oval), margin (well-defined vs. ill-defined), internal echogenicity (homogeneous vs. heterogeneous), presence of cystic components (cystic-solid/patchy anechoic areas vs. solid/sponge-like anechoic areas), calcifications (absent vs. present), and posterior acoustic enhancement (absent vs. present). Representative US images and corresponding characteristics are provided in **Supplementary Figure 1**. In cases of discordant interpretation, the consensus of two raters was adopted as the final assessment. Inter-observer agreement for ultrasound features was assessed using Fleiss’ Kappa.

### 2.3 Image segmentation

All images were converted to NIfTI (.nii.gz) format and resampled to an isotropic voxel spacing of 1×1×1 mm³. Regions of interest (ROI) were manually delineated along the tumor margins using ITK-SNAP software (version 3.8.0). All ROIs were initially segmented by a physician (Reader D) with 5 years of experience in superficial organ US. After a 4-week washout period, 100 randomly selected cases were independently re-delineated by Readers D and E (5 years of experience). Intraclass correlation coefficient (ICC) was used to assess intra- and inter-observer agreement, with ICC≥0.75 indicating good consistency.

### 2.4 Radiomics model construction

Radiomic features were extracted in the training set using PyRadiomics (version 3.0.1) in compliance with the Imaging Biomarker Standardisation Initiative (IBSI) guidelines (24). Three categories of quantitative features were obtained: (1) morphological features, describing the geometric properties of the lesion; (2) first-order features, reflecting the distribution of voxel intensities; and (3) texture features, quantifying intratumoral spatial gray-level relationships and intensity variations, including gray-level co-occurrence matrix (GLCM), gray-level run-length matrix (GLRLM), gray-level size zone matrix (GLSZM), and neighborhood gray-tone difference matrix (NGTDM).

For each nodule, radiomic features extracted from long-axis and short-axis US images were fused at the early feature level to generate a unified feature set that integrated complementary spatial and morphological information from both imaging planes. The radiomics features showed strong reproducibility: intra-observer ICCs ranged from 0.82 to 0.99 (median: 0.94); inter-observer ICCs ranged from 0.78 to 0.98 (median: 0.91)—all ≥ 0.75, indicating good reliability. In the feature selection process, all features were first standardized using Z-score normalization and screened for statistical significance using t-tests (*P* < 0.05). Subsequently, Pearson correlation coefficients were calculated to assess inter-feature redundancy, and features with correlation coefficients≥0.9 were removed (one feature retained per highly correlated group). The maximum relevance minimum redundancy (mRMR) algorithm was then applied to further reduce redundancy and mitigate overfitting. Finally, least absolute shrinkage and selection operator (LASSO) logistic regression was employed with 10-fold cross-validation to select the most discriminative feature subset by tuning the regularization parameter λ according to the minimum error criterion.

The selected features were input into multiple classifiers, including support vector machine (SVM), RandomForest, ExtraTrees, extreme gradient boosting (XGBoost), and light gradient boosting machine (LightGBM). Hyperparameters were optimized via grid search combined with 5-fold cross-validation on the training set to identify the most stable configuration with optimal generalization performance. Detailed hyperparameter settings are provided in **Supplementary Material 1**.

### 2.5 DL model construction

During data preparation, images were cropped and transformed into the minimum bounding rectangle enclosing ROI to eliminate irrelevant background information. All images were standardized using Z-score normalization to standardize intensity distributions. Data augmentation was applied during training, including random cropping and horizontal/vertical flipping; only normalization was performed during validation and testing. Transfer learning was implemented using pre-trained convolutional neural networks (ResNet18, ResNet50, ResNet101, DenseNet121, and DenseNet201), which were fine-tuned for the binary classification task. Hyperparameters were optimized via cross-validation to ensure stable convergence and optimal predictive performance; detailed configurations are provided in **Supplementary Material 2**.

(1) To leverage complementary spatial information from different US views, long-axis and short-axis images were fused to construct a three-channel composite input, forming the Combined Model. The fusion scheme is defined as:


$$\text{Image}=\left[Slice_{long},Slice_{short},\frac{Slice_{long}+Slice_{short}}{2}\right]$$

This strategy integrates morphological and spatial features from both orientations, enhancing the model’s ability to capture comprehensive lesion characteristics. (2) For individual analysis of long-axis and short-axis views, each single-channel image was replicated three times to generate a pseudo-three-channel input, ensuring compatibility with pre-trained (convolutional neural network, CNN) architectures while preserving the intrinsic spatial structure of each view. (3) To compare the impact of different fusion strategies on model performance, a Feature Fusion Model was also constructed by fusing deep features before decision-making: deep features (1920-dimensional and 2048-dimensional, respectively) were extracted from the average pooling layer of the Long-axis and Short-axis Models. Each was reduced to 63 dimensions using principal component analysis (PCA), then concatenated and fed into the traditional classifier for final classification.

### 2.6 Clinical model and nomogram construction

Clinical characteristics and US features of PA and WT were compared. Univariate and multivariate analyses were performed to identify potential clinical predictors, with statistical significance defined as *P* < 0.05. A clinical prediction model was developed based on the identified independent predictors. Furthermore, a Nomogram incorporating the most discriminative radiomics and DL model with clinical variables was constructed using logistic regression, establishing a comprehensive predictive framework.

### 2.7 Physicians’ subjective assessment and AI–assisted diagnosis

Two rounds of subjective evaluation were conducted to assess the diagnostic assistance performance of the AI model. First, three US physicians (A, B and C) independently diagnosed cases in the testing set without knowledge of the pathological results. The physicians had access to relevant clinical information and thoroughly reviewed both long-axis and short-axis US images to simulate real-world diagnostic conditions. After a 4-week washout period, the same physicians re-evaluated the test set with the best-performing AI model assistance in a randomized order. During the second round, they were provided with the model’s prediction outputs, including the probabilities for PA and WT with 0.5 as the probability threshold. The clinical value of the AI model was evaluated by comparing diagnostic performance before and after model assistance.

### 2.8 Statistical analysis

For categorical variables, the chi-square test was used for both two-group and three-group comparisons. For continuous variables, normality was assessed using the Shapiro-Wilk test and homogeneity of variances using Levene’s test. Normally distributed continuous variables were expressed as mean ± SD and compared using the two-sample t-test for two-group comparisons or one-way ANOVA for three-group comparisons. Non-normally distributed continuous variables were expressed as median (IQR) and compared using the Mann-Whitney U test for two-group comparisons or the Kruskal-Wallis H test for three-group comparisons. All tests were two-tailed, with *P* < 0.05 considered statistically significant.

Model discrimination was evaluated using receiver operating characteristic (ROC) curves, with performance quantified by the area under the curve (AUC), accuracy, sensitivity, and specificity. DeLong test was employed for pairwise comparisons of AUCs among multiple models. Calibration curves were used to assess the agreement between predicted probabilities and observed outcomes, and decision curve analysis (DCA) was performed to evaluate the model’s potential clinical utility.

Data analysis and statistical computations were conducted using Python (v3.7.12) and Statsmodels (v0.13.2). Radiomic features were extracted via PyRadiomics (v3.0.1), and deep learning models were implemented in PyTorch (v1.11.0) with computational acceleration provided by CUDA (v11.3.1) and cuDNN (v8.2.1).

## 3 Results

### 3.1 Baseline characteristics and clinical model analysis

A total of 656 patients were included in this study, with 556 enrolled at Center 1 (339 males and 217 females; age range: 14–87 years; mean age: 52.48 ± 14.44 years) and 100 at Center 2 (65 males and 35 females; age range: 16–78 years; mean age: 52.34 ± 15.89 years). The training cohort comprised 215 cases of PA and 174 cases of WT, the internal validation cohort included 87 PA and 80 WT, and the external validation cohort contained 59 PA and 41 WT.

Baseline clinical and US characteristics are summarized in **Table 1**. The Fleiss’ Kappa values were: shape, 0.820 (95% CI: 0.788-0.852); margin, 0.840 (95% CI: 0.809-0.871); echogenicity, 0.815 (95% CI: 0.783-0.847); cystic components, 0.792 (95% CI: 0.760-0.824); calcifications, 0.823 (95% CI: 0.792-0.854); and posterior acoustic enhancement, 0.715 (95% CI: 0.683-0.747), indicating substantial agreement. The three-group comparisons demonstrated that age, gender, lesion diameter, cystic components, calcification, and posterior acoustic enhancement were balanced across cohorts (*P*>0.05), indicating comparability of demographic and key ultrasound features between centers. The primary differences are manifested in smoking and drinking history, likely reflecting population heterogeneity across centers and the limited sample size of the external test set. Notably, the training and internal validation sets (both from Center 1) remained comparable across all variables (*P*>0.05), confirming the validity of the random split. Univariate analysis revealed that gender, smoking history, drinking history, tumor shape, margin definition, cystic components, and posterior acoustic enhancement differed significantly between PA and WT (*P* < 0.05). Multivariate analysis identified male gender (OR = 0.375), smoking history (OR = 17.362), round/oval tumor shape (OR = 0.115), solid/sponge-like anechoic areas (OR = 3.352) as independent predictors of WT (**Supplementary Table 1**).

**Table 1:** Baseline clinical and US characteristics of patients in training, validation and testing sets.

Clinical and US characteristics	Training set	Validation set	Testing set	*P* (Train vs Val)	*P* (Three groups)
Age	52.74 ± 14.62	51.90 ± 14.04	52.34 ± 15.89	0.456	0.824
Maximum diameter	2.83 ± 1.01	2.95 ± 0.99	2.67 ± 0.92	0.142	0.086
Gender				0.75	0.686
Male	235(60.41)	104(62.28)	65(65.00)		
Female	154(39.59)	63(37.72)	35(35.00)		
Smoking				0.81	<0.001
Absent	213(54.76)	94(56.29)	17 (17.00)		
Present	176(45.24)	73(43.71)	83 (83.00)		
Drinking				0.136	<0.001
Absent	249(64.01)	95(56.89)	26(26.00)		
Present	140(35.99)	72(43.11)	74(74.00)		
Shape				0.701	0.002
Round/oval	312(80.21)	137(82.04)	95(95.00)		
Irregular/lobulated	77(19.79)	30(17.96)	5(5.00)		
Margin				0.536	0.004
Well-defined	340(87.40)	142(85.03)	98(98.00)		
Poorly-defined	49(12.60)	25(14.97)	2(2.00)		
Echogenicity				0.179	0.038
Homogeneous	70(17.99)	39(23.35)	29(29.00)		
Heterogeneous	319(82.01)	128(76.65)	71(71.00)		
Cystic components				0.755	0.700
Cystic-solid/patchy anechoic areas	325(83.55)	137(82.04)	86(86.00)		
Solid/sponge-like anechoic areas	64(16.45)	30(17.96)	14(14.00)		
Calcification				0.366	0.323
Absent	350(89.97)	155(92.81)	94(94.00)		
Present	39(10.03)	12(7.19)	6(6.00)		
Posterior acoustic enhancement				1.0	0.984
Absent	77(19.79)	33(19.76)	19(19.00)		
Present	312(80.21)	134(80.24)	81(81.00)		

US, ultrasound. Note-numbers in parentheses are percentages.

Numerical encoding of the aforementioned features was performed, and predictive models were developed using multiple machine learning algorithms. The diagnostic performance of all Clinical Models is summarized in **Supplementary Table 2**, **Supplementary Figure 2**. The ExtraTrees model achieved the highest and most stable predictive performance across the training (AUC: 0.856), internal validation (AUC: 0.843), and external test (AUC: 0.770) cohorts. In the external test set, the ExtraTrees model also achieved the highest accuracy (0.740), with a sensitivity of 0.951.

### 3.2 Performance of radiomics model

**Figure 3:**
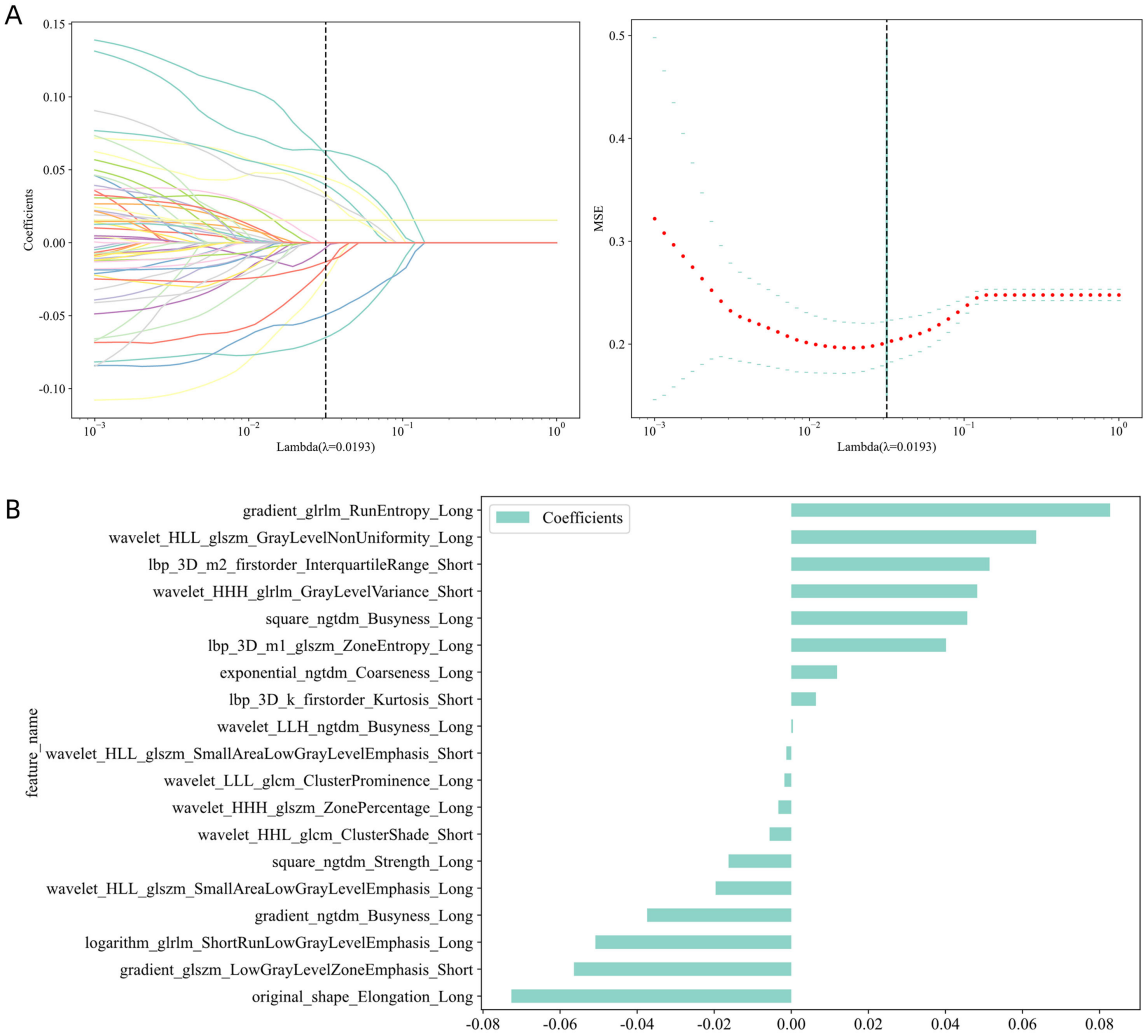
**(A)** Feature selection using LASSO through ten-fold cross-validation and corresponding coefficients. The optimal λ value of 0.0193 was selected. **(B)** Of the 19 radiomic features selected, 13 were from the long-axis view. LASSO, least absolute shrinkage and selection operator.

In this study, radiomics features were extracted from both the long-axis and short-axis views of tumors, with 1562 features obtained from each plane. These were concatenated at the feature level to form a fused set of 3124 features. This integrative approach captured complementary morphological and textural information from two orthogonal perspectives, thereby enhancing discriminative capacity for differential diagnosis. The distribution of feature types is presented in **Supplementary Figure 3**. Through sequential feature selection (t-test: 1395 features; Pearson correlation: 373 features; mRMR: 64 features; LASSO regression), 19 most informative handcrafted features were retained for constructing the conventional radiomics model, of which 13 originated from the long-axis view, indicating its greater contribution to model performance (**Figure 3**). Model predictive performance is summarized in **Supplementary Table 3**, **Supplementary Figure 4**. In the training cohort, the SVM model achieved an accuracy of 0.817 and an AUC of 0.865 (95% CI: 0.828-0.902), with well-balanced sensitivity and specificity (0.776 and 0.851, respectively). The model demonstrated robust performance in the internal validation cohort, yielding an AUC of 0.810 (95% CI: 0.745-0.875) and accuracy of 0.754. In the testing cohort, the model’s performance showed a notable decline in AUC to 0.666 (95% CI: 0.555-0.776), yet it retained acceptable sensitivity (0.683) and specificity (0.678).

### 3.3 Performance of DL model

DL model performance comparisons are presented in **Supplementary Tables 4**-**7**, **Supplementary Figures 5**-**8**. Among single-plane models, DenseNet201 achieved the highest validation AUC (0.827) for the long-axis view, with an accuracy of 0.766 and sensitivity of 0.812, and maintained an AUC of 0.793 in the external test set. For the short-axis view, ResNet50 achieved the best validation AUC (0.857), with accuracy, sensitivity, and specificity of 0.790, 0.762, and 0.816, respectively, and an AUC of 0.820 in the external test set.

Among the Feature Fusion Models based on dual-plane deep features, ExtraTrees achieved a validation AUC of 0.863, comparable to XGBoost (also 0.863), but demonstrated superior generalization in the external test set with an AUC of 0.818 and accuracy of 0.770, compared to 0.805 and 0.760 for XGBoost. The sensitivity and specificity of ExtraTrees were 0.805 and 0.746, respectively. Based on its better cross-center generalizability, ExtraTrees was selected as the optimal classifier. In the Combined Models using fused long- and short-axis images, DenseNet201 outperformed other models, with an AUC of 0.905, accuracy of 0.832, and high specificity of 0.954 in validation cohort. In the external test cohort, it achieved an AUC of 0.860, with sensitivity and specificity of 0.805 and 0.763, respectively.

### 3.4 Performance of comprehensive model and comparison of different models

**Figure 4:**
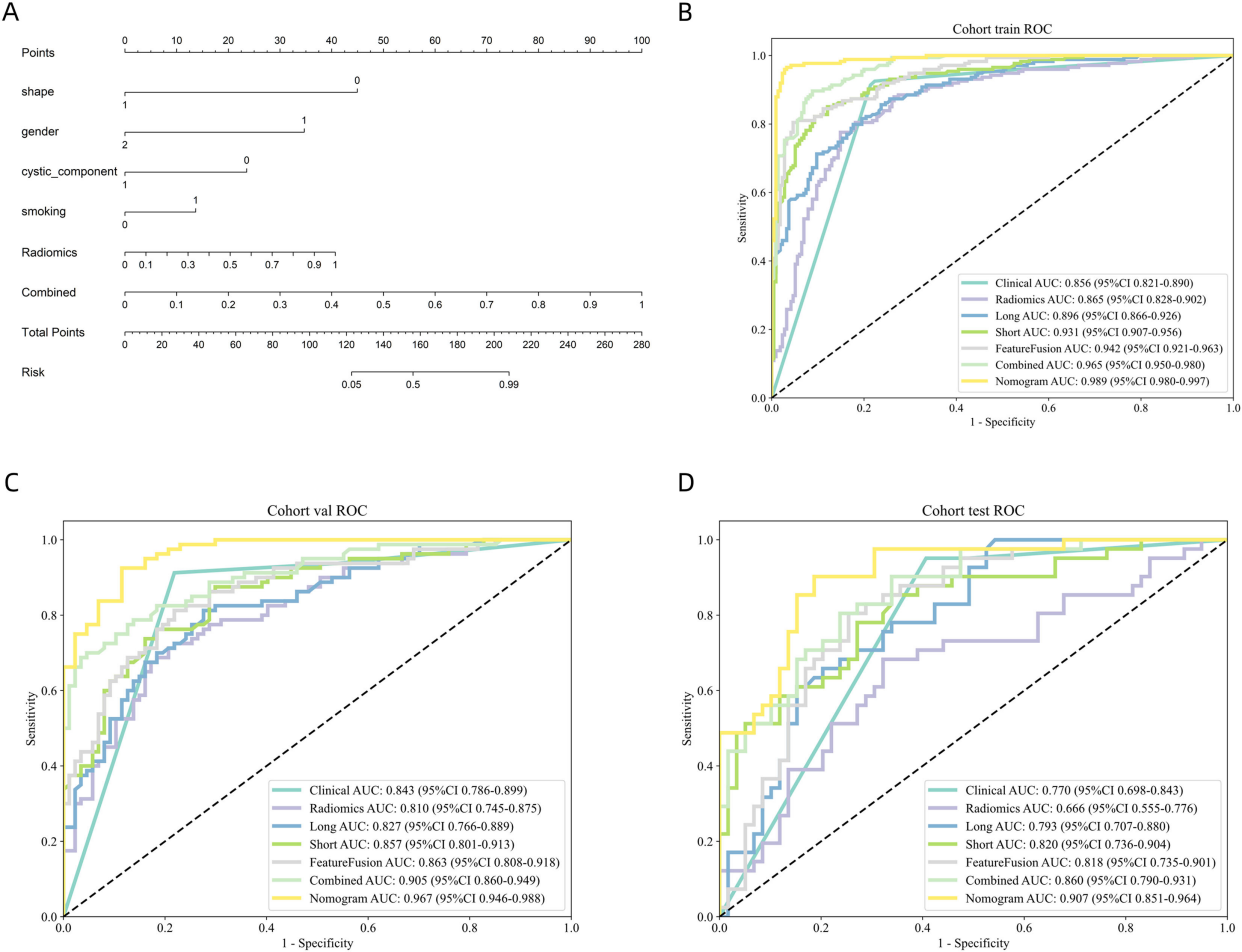
The Nomogram **(A)** for clinical use and ROC curves for different models in training **(B)**, validation **(C)**, and testing **(D)** sets. ROC, receiver operating characteristic. For the Nomogram **(A)**, clinical features are coded as follows: shape (1=irregular, 0=regular); gender (2=female, 1=male); cystic component (1= cyst-predominant/large anechoic areas, 0= solid-predominant/sponge-like anechoic areas); smoking history (0=absent, 1=present).

**Table 2** summarizes the performance of the Clinical Model, Radiomics Model, and DL Models with various fusion strategies. As shown in **Figures 4B–D**, the image-level Combined DL Model achieved the highest performance in the training cohort, with an AUC of 0.965 (95% CI: 0.950-0.980) and an accuracy of 0.905. The model maintained robust generalizability in the internal validation cohort (AUC: 0.905, 95% CI: 0.860-0.950) and the external test cohort (AUC: 0.860, 95% CI: 0.790-0.931), outperforming single-view ResNet50 models (long-axis or short-axis only) and the dual-view Feature Fusion Model. In contrast, the Clinical and Radiomics Models exhibited lower discriminative ability, with validation cohort AUCs of 0.843 and 0.810, and test cohort AUCs of 0.770 and 0.666, respectively.

**Table 2:** Model performance of different models in the training, validation, and testing sets.

Model	Accuracy	AUC	95% CI	Sensitivity	Specificity	PPV	NPV
Clinical-training	0.843	0.856	0.821 - 0.890	0.925	0.777	0.770	0.928
Radiomics-training	0.817	0.865	0.828 - 0.901	0.776	0.851	0.808	0.824
Long-training	0.812	0.896	0.866 - 0.926	0.799	0.823	0.785	0.835
Short-training	0.866	0.931	0.907 - 0.955	0.851	0.879	0.851	0.879
Feature Fusion-training	0.887	0.942	0.920 - 0.963	0.805	0.953	0.933	0.858
Combined-training	0.905	0.965	0.950 - 0.980	0.897	0.912	0.891	0.916
Nomogram-training	0.967	0.989	0.980 - 0.997	0.966	0.967	0.960	0.972
Clinical-validation	0.844	0.843	0.786 - 0.899	0.912	0.782	0.793	0.907
Radiomics-validation	0.754	0.810	0.744 - 0.874	0.725	0.782	0.753	0.756
Long-validation	0.766	0.827	0.765 - 0.888	0.812	0.724	0.730	0.808
Short-validation	0.790	0.857	0.800- 0.912	0.762	0.816	0.792	0.789
Feature Fusion-validation	0.796	0.863	0.807 - 0.918	0.812	0.782	0.774	0.819
Combined-validation	0.832	0.905	0.859 - 0.949	0.700	0.954	0.933	0.776
Nomogram-validation	0.904	0.967	0.945 - 0.988	0.925	0.885	0.881	0.928
Clinical-testing	0.740	0.770	0.697 - 0.842	0.951	0.593	0.619	0.946
Radiomics-testing	0.680	0.666	0.554 - 0.776	0.683	0.678	0.596	0.755
Long-testing	0.680	0.793	0.706 - 0.879	1.000	0.458	0.562	1.000
Short-testing	0.740	0.820	0.735 - 0.904	0.854	0.661	0.636	0.867
Feature Fusion-testing	0.770	0.818	0.734 - 0.900	0.805	0.746	0.687	0.846
Combined-testing	0.780	0.860	0.789 - 0.930	0.805	0.763	0.702	0.849
Nomogram-testing	0.850	0.907	0.850 -0.964	0.902	0.814	0.771	0.923

AUC, area under the curve; CI, confidence interval; PPV, positive predictive value; NPV, negative predictive value.

The Nomogram integrating deep learning, radiomics, and clinical US predictors (**Figure 4A**) further improved diagnostic performance across all cohorts. It achieved an accuracy of 0.967 and an AUC of 0.989 (95% CI: 0.980-0.997) in the training cohort, and an accuracy of 0.904 and an AUC of 0.967 in the validation cohort. In the external testing cohort, while other models showed varying degrees of performance degradation, the Nomogram maintained high sensitivity (0.902), specificity (0.814), and accuracy (0.850), and its AUC remained at a high level of 0.907 (95% CI: 0.850-0.964), indicating that multimodal integration effectively enhances model robustness and predictive efficacy. DeLong test (**Supplementary Figure 9**) showed that the Nomogram’s AUC was significantly higher than all other models in both training and validation sets (*P* < 0.05), but not significantly different from the image-level Combined Model in the external test cohort (*P*>0.05). Calibration curves (**Supplementary Figure 10**) demonstrated excellent agreement between predicted and observed outcomes, and DCA (**Supplementary Figure 11**) confirmed superior clinical net benefit across a wide range of threshold probabilities.

### 3.5 Diagnostic performance of physicians and AI–assisted diagnosis

**Figure 5:**
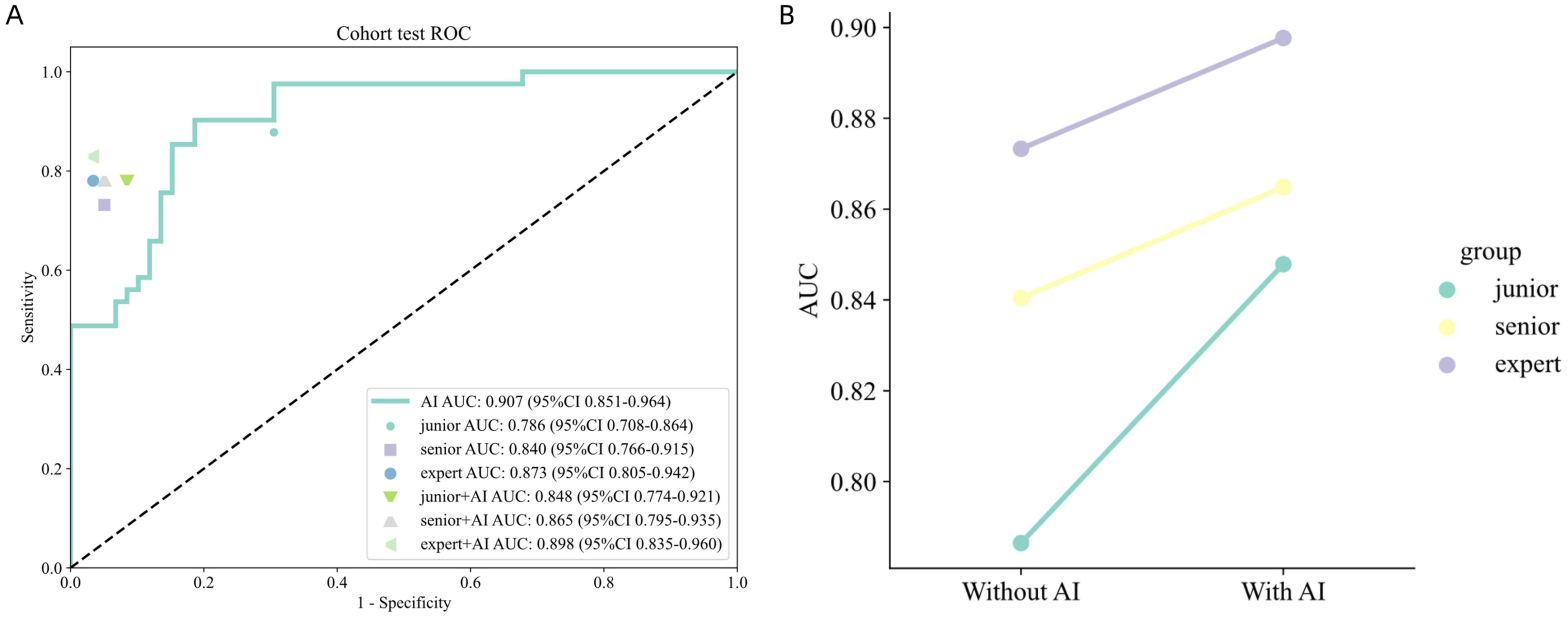
**(A)** Feature selection using LASSO through ten-fold cross-validation and corresponding coefficients. The optimal λ value of 0.0193 was selected. **(B)** Of the 19 radiomic features selected, 13 were from the long-axis view. LASSO, least absolute shrinkage and selection operator.

The diagnostic performance of US physicians with varying experience levels in differentiating PA from WT is summarized in **Table 3**. In the first-round independent evaluation, expert C achieved higher accuracy (0.89) and AUC (0.873) than senior physician B (0.86 and 0.840) and junior physician A (0.77 and 0.786), respectively, although all three physicians performed below the optimal AI model (AUC: 0.907). In the second round with Nomogram assistant, diagnostic performance improved across all physicians. Notably, junior physician A showed significant improvement with model assistance: AUC increased by 0.062, and specificity rose from 0.695 to 0.915, indicating a marked enhancement in diagnostic precision. For senior physician B and expert C, sensitivity improved (0.732 to 0.780 and 0.780 to 0.829, respectively), while high baseline specificity and positive predictive value were maintained. This led to increased accuracy (from 0.86 to 0.88 for physician B and from 0.89 to 0.91 for expert C), resulting in more balanced and reliable diagnostic decisions. **Figure 5** illustrates the changes in subjective assessments across the two rounds. The greatest AUC improvement was observed in junior physician A, while expert C achieved the best overall performance under model guidance (accuracy: 0.91, AUC: 0.898).

**Table 3:** Diagnostic performance of physicians and AI–assisted diagnosis.

Signature	Accuracy	AUC	95% CI	Sensitivity	Specificity	PPV	NPV
AI	0.85	0.907	0.850 - 0.964	0.902	0.814	0.771	0.923
Junior	0.77	0.786	0.708 - 0.864	0.878	0.695	0.667	0.891
Senior	0.86	0.840	0.766 - 0.914	0.732	0.949	0.909	0.836
Expert	0.89	0.873	0.805 - 0.941	0.780	0.966	0.941	0.864
Junior + AI	0.86	0.848	0.774 - 0.921	0.780	0.915	0.865	0.857
Senior + AI	0.88	0.865	0.794 - 0.934	0.780	0.949	0.914	0.862
Expert + AI	0.91	0.898	0.834 - 0.960	0.829	0.966	0.944	0.891

AI, artificial intelligence; AUC, area under the curve; CI, confidence interval; PPV, positive predictive value; NPV, negative predictive value.

## 4 Discussion

This study developed and validated a dual-plane integrated model based on US images for accurate differentiation of PA from WT. By leveraging complementary information from two orthogonal imaging planes, the proposed approach effectively overcomes the limitations of conventional single-view diagnostic strategies. The Nomogram integrating clinical US characteristics, dual-plane radiomics, and image fusion-based DL demonstrated superior diagnostic performance, with significantly higher AUC values compared to models using single-view or single-feature inputs. Moreover, the model achieved diagnostic performance surpassing that of junior physicians in independent assessments and served as an effective decision-support tool to improve their diagnostic consistency. These findings indicate that the dual-plane comprehensive model holds substantial clinical value in enhancing diagnostic precision for PA and WT, facilitating optimized patient management and reducing unnecessary surgical interventions.

PA and WT exhibit overlapping clinical manifestations; however, they differ markedly in biological behavior, therapeutic management, and prognosis. PA consists of epithelial, myoepithelial, and stromal components, and is histologically classified into mixed, mucinous, and cellular subtypes based on stromal proportion (25). Surgical excision remains the primary treatment; however, lesions with high-risk capsular features—such as incomplete capsule, infiltration, pseudopodia, or satellite nodules—as well as those with mucinous histology, are prone to intraoperative rupture, residual disease, and recurrence (26–28). Reported 5-year recurrence rates range from 1.6% to 23%, with malignancy risk increasing over time (1.5% at 5 years, rising to 9.5% at 15 years) (26, 29). In contrast, Warthin tumor is almost exclusively parotid in origin and represents the second most common benign parotid neoplasm, predominantly affecting middle-aged and elderly male smokers. Notably, the male predominance has been diminishing in recent years due to rising smoking rates among women (30). Given its indolent course, minimal symptoms, and frequent occurrence in older adults, active surveillance is increasingly considered a viable management option. Surgical intervention is reserved for cases with diagnostic uncertainty, cosmetic concerns, symptomatic progression (e.g., pain, ulceration, infection), or strong patient preference (8).

The subjectivity in medical image interpretation underscores the urgent need for improved preoperative diagnostic tools for parotid tumors. He et al. (22) developed an integrated machine learning model using multicenter data by combining clinical, US, and radiomics features, achieving an AUC of 0.833 on the test set—superior to both single radiomics and clinical models, and significantly outperforming clinicians (AUC: 0.681). In recent years, advances in computational power and the availability of large-scale datasets have propelled deep learning into a major research focus. Liu et al. (31) evaluated five deep learning models for differentiating PA from WT on US images, with ResNet50 yielding the highest performance (AUC: 0.908). All deep learning models surpassed both radiologists and FNAC in diagnostic accuracy. In our study, the DenseNet201 and ResNet50 models based on single−plane input demonstrated strong diagnostic performance and generalization ability, significantly outperforming the conventional radiomics model. Meanwhile, the performance of the radiomics model decreased notably in the external test cohort, with the AUC dropping from 0.865 to 0.666. This decline may be attributed to the fact that most radiomics features are low−level handcrafted features, which are sensitive to inter−device variability and image acquisition parameters. In contrast, the deep learning model is capable of extracting high−level semantic features, conferring greater robustness and generalization ability, thereby maintaining relatively stable performance in the external test cohort.

A recent systematic review (32) compared the performance of radiomics, deep learning, and multimodal fusion models in medical image analysis. The results showed that fusion models outperformed others in 63% of the included studies, underperformed in 25%, and were comparable in 13%. This indicates that different feature types have distinct advantages and limitations, and integrating multiple features may enable more comprehensive capture of diagnostic information. In our previous study (17), a nomogram model combining clinical US features with radiomics and deep learning features achieved the best performance in differentiating benign from malignant parotid tumors (AUC: 0.934). Similarly, Mao et al. (23) developed a DLRN model by integrating radiomics, deep learning, and clinical features to distinguish pleomorphic adenoma from Warthin tumor, achieving an AUC of 0.908 in both internal and external validation cohorts, demonstrating strong generalization ability.

However, conventional US-based radiomics or deep learning studies have predominantly focused on single-plane analysis. Fusing multi-planar images has been shown to provide complementary spatial and morphological information, thereby improving model predictive performance (33–35). US imaging is inherently dynamic and multi-view. This study adopts dual-plane image analysis to effectively correct the sampling bias of a single plane by increasing feature space diversity and spatial sampling density. The measurable differences in radiomics and DL features across different planes further enhance the model’s classification and discrimination capability, making automated analysis more consistent with real clinical practice.

For fusing multi-modal or multi-section data, common strategies include early, mid-level, and late fusion. In the field of MRI, Sunnetci et al. (36) employed discrete wavelet transform (DWT) for image fusion across different sequences and compared the performance of three architectures: ResNet18, GoogLeNet, and DenseNet201. DenseNet201 achieved the best performance in most fusion strategies, with a maximum accuracy of 95.96%, validating the effectiveness of the image fusion. In this study, two deep learning fusion approaches using dual-plane US images were systematically compared: multi-channel image-level fusion (early fusion) and feature-level fusion (mid-level fusion). Image-level fusion concatenates long- and short-axis views along the channel dimension at the input layer to form a multi-channel input, enabling the network to jointly learn co-occurring features from both perspectives at an early stage. In contrast, feature-level fusion employs a dual-branch architecture to extract high-level semantic representations from each view separately, followed by a fusion module to integrate cross-view discriminative patterns. Our results demonstrate that both dual-plane models outperform their single-plane counterparts (long- and short-axis), with the image-level fusion model achieving superior diagnostic accuracy (AUC: 0.860-0.965). This advantage likely stems from the preservation of fine-grained spatial correspondence between planes at the input level, facilitating the learning of critical joint features. In contrast, feature-level fusion loses low-level spatial relationships during hierarchical feature extraction, limiting its discriminative power. Furthermore, we developed a comprehensive model integrating clinical US features, dual-plane radiomics, and deep learning features, which achieved the highest performance in differentiating PA from WT (AUC: 0.907-0.989), demonstrating that multi-source feature fusion effectively overcomes the limitations of single-modality approaches and provides a more robust and complementary basis for diagnostic decision-making.

**Figure 6:**
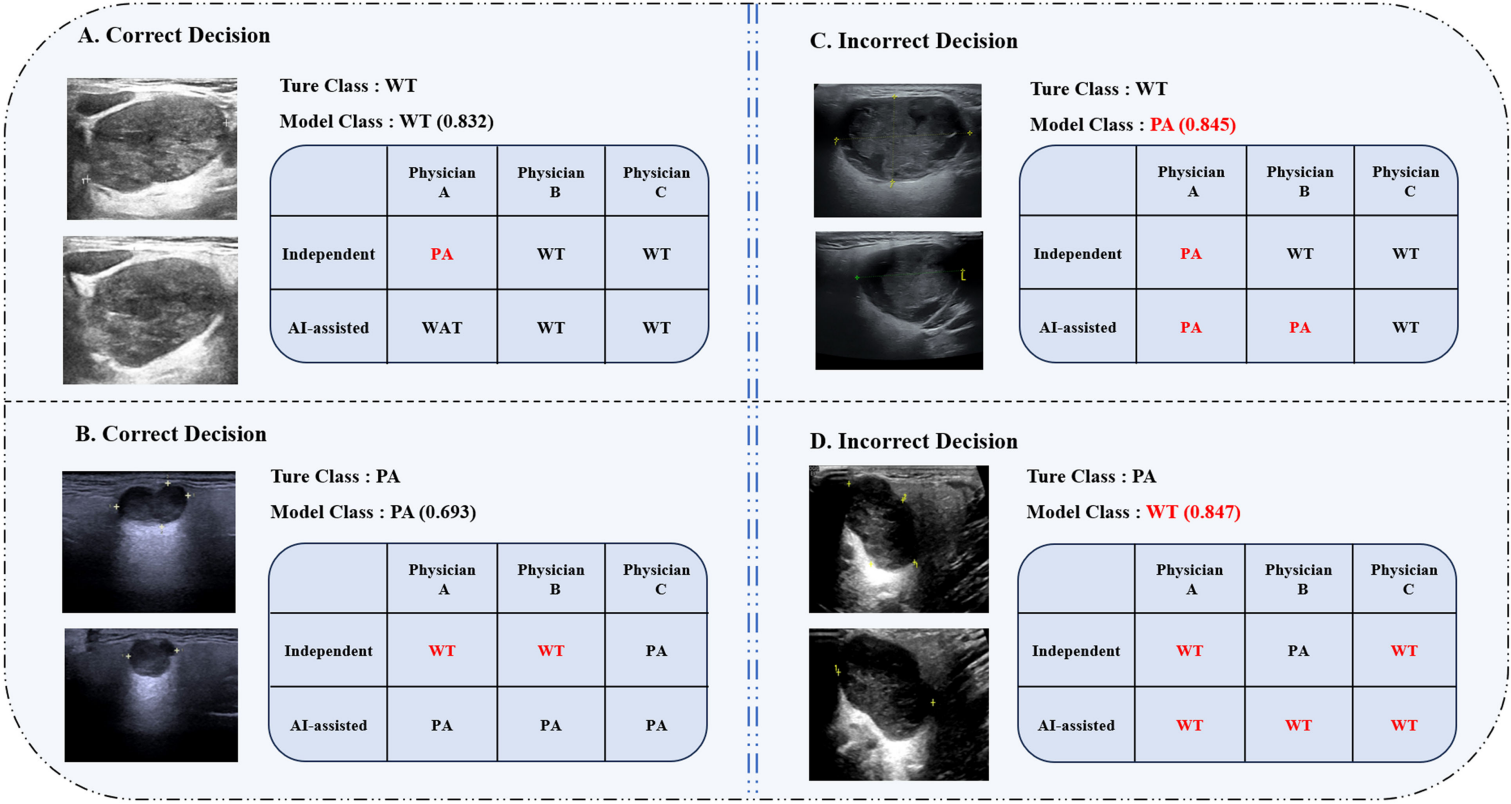
Typical examples of physician diagnosis with and without model assistance. **(A, B)** Correct model decision; **(C, D)** incorrect model decision. AI, artificial intelligence; PA, pleomorphic adenoma; WT, Warthin tumor.

To evaluate the clinical utility of the comprehensive model, we compared the diagnostic performance of three US physicians with varying levels of experience in the presence and absence of model assistance. **Figure 6** shows several typical examples of model-assisted diagnosis. Results showed that all three physicians achieved higher AUC values when aided by the model. Notably, the junior physician exhibited the most substantial improvement, with final accuracy and AUC approaching those of the senior physician. Moreover, the model further enhanced the sensitivity and accuracy of both the senior and expert physicians, demonstrating its consistent value across different experience levels. It should be noted that clinical decision support systems are intended to augment, not replace, clinical judgment. In this study, the core value of AI assistance lies in improving the diagnostic performance of junior physicians, narrowing the gap with senior physicians, and thereby reducing unnecessary biopsies or surgeries. Furthermore, the superior performance of the standalone AI model suggests its potential value as a second reader or quality control tool.

Certainly, this study has several limitations. First, the retrospective design may introduce selection bias. Second, manual segmentation inevitably carries a risk of inter-observer variability, which may affect the stability of radiomics features, particularly morphological features, thereby potentially influencing model performance. Future work will explore semi- or fully automated deep learning-based segmentation methods to further enhance the reproducibility and clinical utility of the proposed model. Third, although dual-plane imaging substantially improved model performance compared to conventional single-plane approaches, it remains insufficient to fully capture the spatial heterogeneity and dynamic characteristics of tumors. Future studies may explore multi-frame modeling based on US video sequences to better emulate real-world clinical assessment. Fourth, only image−level and feature−level fusion were evaluated among fusion strategies; the potential benefits of late decision fusion and other integration methods warrant further investigation. Finally, multimodal imaging data such as elastography or contrast−enhanced US were not incorporated in this study. In future work, the sample size will be expanded, cross−modal data including CT and MRI will be integrated, and the model’s generalizability will be validated across multiple external independent cohorts and prospective trials.

## 5 Conclusions

The proposed dual−plane US−based comprehensive model shows promising performance in differentiating PA from WT, potentially providing decision support for clinicians and helping to reduce diagnostic variability associated with operator experience levels. This approach warrants further exploration for the clinical implementation of precision diagnosis and treatment in parotid tumor management.

## Data Availability

The original contributions presented in the study are included in the article/**Supplementary Material**. Further inquiries can be directed to the corresponding author.
