# Supplementary material for: A comprehensive model with dual plane ultrasound based deep learning radiomics to distinguish parotid pleomorphic adenoma from Warthin tumor
Source: Front Oncol. 2026 Jul 24;16:1890152. doi: 10.3389/fonc.2026.1890152 (PMC13447068; doi:10.3389/fonc.2026.1890152)
Supplement: Supplementary file 1 [file DataSheet1.docx]

Supplementary Material

# 1 Details of Hyperparameter Configurations for Machine Learning Algorithms in Radiomics Models

**SVC:** RBF kernel, probability=True, max_iter=144, C=0.8, random_state=0;

**LightGBM:** n_estimators=7, max_depth=2;

**Random Forest:** n_estimators=7, max_depth=3;

**ExtraTrees:** n_estimators=30, max_depth=2, min_samples_split=2;

**XGBoost:** n_estimators=7, max_depth=2, min_child_weight=2, objective='binary:logistic', use_label_encoder=False, eval_metric='error'.

# 2 Details of Deep Learning Training Process

**Data Augmentation:** To enhance model robustness and generalization capability, we implemented real-time data augmentation techniques—including random cropping, horizontal flipping, and vertical flipping—on the training images. These transformations effectively increased intra-dataset variability while preserving the structural integrity of the visual content. Z-score normalization was applied to standardize the intensity distribution across all RGB channels, thereby ensuring consistent input representation for the model. For the validation and test datasets, only normalization was performed to maintain an unbiased and uniform evaluation protocol.

**Data Normalization:** Grayscale values of image slices were standardized using min-max normalization, scaling pixel intensities to the range of [−1, 1]. This normalization strategy facilitated uniform data processing and improved learning efficiency during model training. Each cropped subregion was resized to a fixed dimension of 224 × 224 pixels using nearest-neighbor interpolation, ensuring compatibility with the input specifications of the underlying neural network architectures.

**Training Parameters:** A transfer learning approach was employed, with model initialization based on pre-trained weights from the ImageNet Large Scale Visual Recognition Challenge 2012 (ILSVRC-2012). To optimize training dynamics and mitigate the risk of premature convergence, a cosine decay strategy was adopted, formulated as follows:

$$\boldsymbol{\eta}_{\boldsymbol{t}}=\boldsymbol{\eta}_{\boldsymbol{min}}^{\boldsymbol{i}}+\frac{\mathbf{1}}{\mathbf{2}}\left( \boldsymbol{\eta}_{\boldsymbol{max}}^{\boldsymbol{i}}-\boldsymbol{\eta}_{\boldsymbol{min}}^{\boldsymbol{i}} \right)\left( \mathbf{1}+\mathbf{cos}\left( \frac{\boldsymbol{T}_{\boldsymbol{cur}}}{\boldsymbol{T}_{\boldsymbol{i}}}\boldsymbol{\pi} \right) \right) \text{(2)}$$

where $\eta_{min}^{i}$ is set to 0, $\eta_{max}^{i}$ is set to 0.01, and $T_{i}$ represents the total number of training epochs, set at 96.

- **Optimizer:** Stochastic Gradient Descent (SGD) was chosen for its well-documented efficacy in handling noisy gradient signals., which enhances stability and maintains compatibility across various network architectures.
- **Loss Function:** We used softmax cross-entropy as the loss metric to enable multi-class classification by providing a probabilistic interpretation of model outputs.
- **Batch Size and Epochs:** Training was conducted with a batch size of 32 over 48 epochs to balance computational efficiency with learning efficacy, thereby promoting better generalization to unseen data.

# 3 Baseline clinical and US characteristics

**Supplementary Figure 1.** Representative US images and corresponding characteristics. **(A)**: oval, well-defined, heterogeneous echo, large anechoic areas, posterior acoustic enhancement. **(B)**: irregular, ill-defined, heterogeneous echo. **(C)**: lobulated, ill-defined, heterogeneous echo, calcifications. **(D)**: oval, well-defined, homogeneous echo. US, ultrasound.

**Supplementary Table 1.** Univariable and multivariable analysis of clinical and US characteristics.

| Clinical and US  characteristics | Univariable Analysis | | | |  | Multivariable Analysis | | | |
| --- | --- | --- | --- | --- | --- | --- | --- | --- | --- |
|  | OR | Lower 95%CI | Upper 95%CI | *P* |  | OR | Lower 95%CI | Upper 95%CI | *P* |
| Shape | 0.116 | 0.063 | 0.214 | <0.05 |  | 0.115 | 0.048 | 0.274 | <0.05 |
| Margin | 0.195 | 0.103 | 0.369 | <0.05 |  | 0.977 | 0.358 | 2.662 | 0.969 |
| Calcification | 0.625 | 0.364 | 1.074 | 0.153 |  |  |  |  |  |
| Gender | 0.639 | 0.566 | 0.722 | <0.05 |  | 0.357 | 0.250 | 0.510 | <0.05 |
| Posterior acoustic enhancement | 0.696 | 0.576 | 0.840 | <0.05 |  | 0.847 | 0.478 | 1.501 | 0.633 |
| Age | 1.002 | 0.999 | 1.005 | 0.281 |  |  |  |  |  |
| Diameter | 1.016 | 0.962 | 1.075 | 0.631 |  |  |  |  |  |
| Echogenicity | 1.019 | 0.848 | 1.225 | 0.867 |  |  |  |  |  |
| Cystic component | 2.048 | 1.322 | 3.174 | <0.05 |  | 3.352 | 1.779 | 6.315 | <0.05 |
| Drinking | 2.256 | 1.669 | 3.050 | <0.05 |  | 0.676 | 0.357 | 1.280 | 0.313 |
| Smoking | 3.513 | 2.606 | 4.735 | <0.05 |  | 17.362 | 9.328 | 32.330 | <0.05 |

US, ultrasound; CI, confidence interval.

# 4 Details of Clinical Models

**Supplementary Table 2.** Model performance of different clinical models in the training, validation, and testing sets.

| Model | Accuracy | AUC | 95% CI | Sensitivity | Specificity | PPV | NPV |
| --- | --- | --- | --- | --- | --- | --- | --- |
| SVM-training | 0.843 | 0.819 | 0.775 - 0.863 | 0.914 | 0.786 | 0.776 | 0.918 |
| SVM-validation | 0.844 | 0.806 | 0.734 - 0.878 | 0.912 | 0.782 | 0.793 | 0.907 |
| SVM-testing | 0.690 | 0.721 | 0.625 - 0.817 | 0.756 | 0.644 | 0.596 | 0.792 |
| RandomForest-training | 0.805 | 0.803 | 0.763 - 0.843 | 0.787 | 0.819 | 0.778 | 0.826 |
| RandomForest-validation | 0.814 | 0.812 | 0.753 - 0.872 | 0.762 | 0.862 | 0.836 | 0.798 |
| RandomForest-testing | 0.550 | 0.615 | 0.554 - 0.676 | 0.976 | 0.254 | 0.476 | 0.937 |
| ExtraTrees-training | 0.843 | 0.856 | 0.821 - 0.890 | 0.925 | 0.777 | 0.770 | 0.928 |
| ExtraTrees-validation | 0.844 | 0.843 | 0.786 - 0.899 | 0.912 | 0.782 | 0.793 | 0.907 |
| ExtraTrees-testing | 0.740 | 0.770 | 0.698 - 0.843 | 0.951 | 0.593 | 0.619 | 0.946 |
| XGBoost-training | 0.807 | 0.822 | 0.787 - 0.856 | 0.960 | 0.684 | 0.711 | 0.955 |
| XGBoost-validation | 0.796 | 0.802 | 0.746 - 0.859 | 0.937 | 0.667 | 0.721 | 0.921 |
| XGBoost-testing | 0.720 | 0.755 | 0.683 - 0.827 | 0.951 | 0.559 | 0.600 | 0.943 |
| LightGBM-training | 0.807 | 0.822 | 0.787 - 0.856 | 0.960 | 0.684 | 0.711 | 0.955 |
| LightGBM-validation | 0.796 | 0.82 | 0.746 - 0.859 | 0.937 | 0.667 | 0.721 | 0.921 |
| LightGBM-testing | 0.720 | 0.755 | 0.683 - 0.827 | 0.951 | 0.559 | 0.600 | 0.943 |

AUC, area under the curve; CI, confidence interval; PPV, positive predictive value; NPV, negative predictive value.

**Supplementary Figure 2.** ROC curves for different clinical models in training **(A)**, validation **(B)** and testing **(C)** sets. ROC, receiver operating characteristic.

# 5 Details of Radiomics Models

**Supplementary Figure 3.** Number and ratio of handcrafted features.

**Supplementary Table 3.** Model performance of different radiomics models in the training, validation, and testing sets.

| Model | Accuracy | AUC | 95% CI | Sensitivity | Specificity | PPV | NPV |
| --- | --- | --- | --- | --- | --- | --- | --- |
| SVM-training | 0.817 | 0.865 | 0.828 - 0.902 | 0.776 | 0.851 | 0.808 | 0.824 |
| SVM-validation | 0.754 | 0.810 | 0.745 - 0.875 | 0.725 | 0.782 | 0.753 | 0.756 |
| SVM-testing | 0.680 | 0.666 | 0.555 - 0.776 | 0.683 | 0.678 | 0.596 | 0.755 |
| RandomForest-training | 0.766 | 0.859 | 0.822 - 0.895 | 0.822 | 0.721 | 0.704 | 0.833 |
| RandomForest-validation | 0.695 | 0.740 | 0.665 - 0.816 | 0.587 | 0.793 | 0.723 | 0.676 |
| RandomForest-testing | 0.700 | 0.672 | 0.561 - 0.783 | 0.683 | 0.712 | 0.622 | 0.764 |
| ExtraTrees-training | 0.774 | 0.828 | 0.787 - 0.869 | 0.799 | 0.753 | 0.724 | 0.822 |
| ExtraTrees-validation | 0.731 | 0.784 | 0.713 - 0.854 | 0.825 | 0.644 | 0.680 | 0.800 |
| ExtraTrees-testing | 0.620 | 0.674 | 0.566 - 0.781 | 0.805 | 0.492 | 0.524 | 0.784 |
| XGBoost-training | 0.799 | 0.889 | 0.857 - 0.921 | 0.885 | 0.730 | 0.726 | 0.887 |
| XGBoost-validation | 0.683 | 0.710 | 0.632 - 0.789 | 0.700 | 0.667 | 0.659 | 0.707 |
| XGBoost-testing | 0.650 | 0.648 | 0.536 - 0.760 | 0.537 | 0.729 | 0.579 | 0.694 |
| LightGBM-training | 0.761 | 0.830 | 0.790 - 0.870 | 0.690 | 0.819 | 0.755 | 0.765 |
| LightGBM-validation | 0.695 | 0.690 | 0.609 - 0.772 | 0.687 | 0.701 | 0.679 | 0.709 |
| LightGBM-testing | 0.590 | 0.615 | 0.502 - 0.728 | 0.756 | 0.475 | 0.500 | 0.737 |

AUC, area under the curve; CI, confidence interval; PPV, positive predictive value; NPV, negative predictive value.

**Supplementary Figure 4.** ROC curves for different radiomics models in training **(A)**, validation **(B)** and testing **(C)** sets. ROC, receiver operating characteristic.

# 6 Details of Long-axis and Short-axis DL Models

**Supplementary Table 4.** Model performance of different Long-axis DL models in the training, validation, and testing sets.

| Model | Accuracy | AUC | 95% CI | Sensitivity | Specificity | PPV | NPV |
| --- | --- | --- | --- | --- | --- | --- | --- |
| Resnet18-training | 0.774 | 0.842 | 0.803-0.881 | 0.684 | 0.847 | 0.783 | 0.768 |
| Resnet18-validation | 0.725 | 0.744 | 0.667-0.820 | 0.725 | 0.724 | 0.707 | 0.741 |
| Resnet18-testing | 0.660 | 0.718 | 0.618-0.817 | 0.829 | 0.542 | 0.557 | 0.821 |
| Resnet50-training | 0.830 | 0.897 | 0.866-0.927 | 0.851 | 0.814 | 0.787 | 0.871 |
| Resnet50-validation | 0.766 | 0.810 | 0.744-0.875 | 0.862 | 0.678 | 0.711 | 0.843 |
| Resnet50-testing | 0.770 | 0.795 | 0.705-0.884 | 0.756 | 0.780 | 0.705 | 0.821 |
| Resnet101-training | 0.784 | 0.881 | 0.849-0.912 | 0.833 | 0.744 | 0.725 | 0.847 |
| Resnet101-validation | 0.743 | 0.798 | 0.730-0.864 | 0.812 | 0.678 | 0.699 | 0.797 |
| Resnet101-testing | 0.740 | 0.754 | 0.658-0.848 | 0.976 | 0.576 | 0.615 | 0.971 |
| Densenet121-training | 0.794 | 0.856 | 0.818-0.893 | 0.822 | 0.772 | 0.745 | 0.843 |
| Densenet121-validation | 0.695 | 0.761 | 0.689-0.831 | 0.775 | 0.621 | 0.653 | 0.750 |
| Densenet121-testing | 0.770 | 0.806 | 0.720-0.891 | 0.537 | 0.932 | 0.846 | 0.743 |
| Densenet201-training | 0.812 | 0.896 | 0.866-0.926 | 0.799 | 0.823 | 0.785 | 0.835 |
| Densenet201-validation | 0.766 | 0.827 | 0.765-0.888 | 0.812 | 0.724 | 0.730 | 0.808 |
| Densenet201-testing | 0.680 | 0.793 | 0.706-0.879 | 1.000 | 0.458 | 0.562 | 1.000 |

DL, deep learning; AUC, area under the curve; CI, confidence interval; PPV, positive predictive value; NPV, negative predictive value.

**Supplementary Figure 5.** ROC curves for different Long-axis DL models in training **(A)**, validation **(B)** and testing **(C)** sets. ROC, receiver operating characteristic; DL, deep learning.

**Supplementary Table 5.** Model performance of different Short-axis DL models in the training, validation, and testing sets.

| Model | Accuracy | AUC | 95% CI | Sensitivity | Specificity | PPV | NPV |
| --- | --- | --- | --- | --- | --- | --- | --- |
| Resnet18-training | 0.730 | 0.800 | 0.755-0.843 | 0.741 | 0.721 | 0.683 | 0.775 |
| Resnet18-validation | 0.731 | 0.778 | 0.709-0.847 | 0.650 | 0.805 | 0.754 | 0.714 |
| Resnet18-testing | 0.710 | 0.741 | 0.642-0.840 | 0.780 | 0.661 | 0.615 | 0.812 |
| Resnet50-training | 0.866 | 0.931 | 0.907-0.955 | 0.851 | 0.879 | 0.851 | 0.879 |
| Resnet50-validation | 0.790 | 0.857 | 0.800-0.912 | 0.762 | 0.816 | 0.792 | 0.789 |
| Resnet50-testing | 0.740 | 0.820 | 0.735-0.904 | 0.854 | 0.661 | 0.636 | 0.867 |
| Resnet101-training | 0.841 | 0.890 | 0.856-0.923 | 0.793 | 0.879 | 0.841 | 0.840 |
| Resnet101-validation | 0.731 | 0.801 | 0.734-0.866 | 0.562 | 0.885 | 0.818 | 0.687 |
| Resnet101-testing | 0.760 | 0.770 | 0.672-0.866 | 0.683 | 0.814 | 0.718 | 0.787 |
| Densenet121-training | 0.748 | 0.814 | 0.771-0.856 | 0.793 | 0.712 | 0.690 | 0.810 |
| Densenet121-validation | 0.760 | 0.802 | 0.734-0.868 | 0.712 | 0.805 | 0.770 | 0.753 |
| Densenet121-testing | 0.720 | 0.802 | 0.713-0.889 | 0.927 | 0.576 | 0.603 | 0.919 |
| Densenet201-training | 0.805 | 0.866 | 0.830-0.901 | 0.736 | 0.860 | 0.810 | 0.801 |
| Densenet201-validation | 0.766 | 0.814 | 0.749-0.879 | 0.812 | 0.724 | 0.730 | 0.808 |
| Densenet201-testing | 0.740 | 0.801 | 0.711-0.889 | 0.902 | 0.627 | 0.627 | 0.902 |

DL, deep learning; AUC, area under the curve; CI, confidence interval; PPV, positive predictive value; NPV, negative predictive value.

**Supplementary Figure 6.** ROC curves for different Short-axis DL models in training **(A)**, validation **(B)** and testing **(C)** sets. ROC, receiver operating characteristic; DL, deep learning.

# 7 Details of Feature Fusion DL Models

**Supplementary Table 6.** Model performance of different Feature Fusion DL models in the training, validation, and testing sets.

| Model | Accuracy | AUC | 95% CI | Sensitivity | Specificity | PPV | NPV |
| --- | --- | --- | --- | --- | --- | --- | --- |
| SVM-training | 0.763 | 0.832 | 0.792 - 0.872 | 0.862 | 0.684 | 0.688 | 0.860 |
| SVM-validation | 0.707 | 0.772 | 0.701 - 0.843 | 0.600 | 0.805 | 0.738 | 0.686 |
| SVM-testing | 0.690 | 0.712 | 0.609 - 0.815 | 0.780 | 0.627 | 0.593 | 0.804 |
| RandomForest-training | 0.874 | 0.946 | 0.926 - 0.967 | 0.845 | 0.898 | 0.870 | 0.877 |
| RandomForest-validation | 0.778 | 0.822 | 0.756 - 0.887 | 0.800 | 0.759 | 0.753 | 0.805 |
| RandomForest-testing | 0.770 | 0.825 | 0.745 - 0.905 | 0.829 | 0.729 | 0.680 | 0.860 |
| ExtraTrees-training | 0.887 | 0.942 | 0.921 - 0.963 | 0.805 | 0.953 | 0.933 | 0.858 |
| ExtraTrees-validation | 0.796 | 0.863 | 0.808 - 0.918 | 0.812 | 0.782 | 0.774 | 0.819 |
| ExtraTrees-testing | 0.770 | 0.818 | 0.735 - 0.901 | 0.805 | 0.746 | 0.687 | 0.846 |
| XGBoost-training | 0.892 | 0.958 | 0.941 - 0.975 | 0.920 | 0.870 | 0.851 | 0.930 |
| XGBoost-validation | 0.820 | 0.863 | 0.806 - 0.920 | 0.800 | 0.839 | 0.821 | 0.820 |
| XGBoost-testing | 0.760 | 0.805 | 0.720 - 0.889 | 0.829 | 0.712 | 0.667 | 0.857 |
| LightGBM-training | 0.874 | 0.934 | 0.910 - 0.957 | 0.856 | 0.888 | 0.861 | 0.884 |
| LightGBM-validation | 0.778 | 0.831 | 0.769 - 0.893 | 0.637 | 0.908 | 0.864 | 0.731 |
| LightGBM-testing | 0.730 | 0.767 | 0.675 - 0.858 | 0.951 | 0.576 | 0.609 | 0.944 |

DL, deep learning; AUC, area under the curve; CI, confidence interval; PPV, positive predictive value; NPV, negative predictive value.

**Supplementary Figure 7.** ROC curves for different Feature Fusion DL models in training **(A)**, validation **(B)** and testing **(C)** sets. ROC, receiver operating characteristic; DL, deep learning.

# 8 Details of Combined DL Models

**Supplementary Table 7**. Model performance of different Combined DL models in the training, validation, and testing sets.

| Model | Accuracy | AUC | 95% CI | Sensitivity | Specificity | PPV | NPV |
| --- | --- | --- | --- | --- | --- | --- | --- |
| Resnet18-training | 0.825 | 0.910 | 0.882-0.937 | 0.845 | 0.809 | 0.782 | 0.866 |
| Resnet18-validation | 0.790 | 0.854 | 0.797-0.910 | 0.887 | 0.701 | 0.732 | 0.871 |
| Resnet18-testing | 0.720 | 0.774 | 0.681-0.866 | 0.780 | 0.678 | 0.627 | 0.816 |
| Resnet50-training | 0.882 | 0.940 | 0.917-0.961 | 0.839 | 0.916 | 0.890 | 0.876 |
| Resnet50-validation | 0.784 | 0.862 | 0.807-0.915 | 0.725 | 0.839 | 0.806 | 0.768 |
| Resnet50-testing | 0.810 | 0.834 | 0.750-0.917 | 0.780 | 0.831 | 0.762 | 0.845 |
| Resnet101-training | 0.856 | 0.923 | 0.896-0.950 | 0.885 | 0.833 | 0.811 | 0.899 |
| Resnet101-validation | 0.778 | 0.854 | 0.797-0.910 | 0.850 | 0.713 | 0.731 | 0.838 |
| Resnet101-testing | 0.820 | 0.835 | 0.751-0.919 | 0.780 | 0.847 | 0.780 | 0.847 |
| Densenet121-training | 0.802 | 0.886 | 0.853-0.918 | 0.839 | 0.772 | 0.749 | 0.856 |
| Densenet121-validation | 0.772 | 0.847 | 0.790-0.903 | 0.650 | 0.885 | 0.839 | 0.733 |
| Densenet121-testing | 0.730 | 0.831 | 0.751-0.909 | 0.951 | 0.576 | 0.609 | 0.944 |
| Densenet201-training | 0.905 | 0.965 | 0.950-0.980 | 0.897 | 0.912 | 0.891 | 0.916 |
| Densenet201-validation | 0.832 | 0.905 | 0.859-0.949 | 0.700 | 0.954 | 0.933 | 0.776 |
| Densenet201-testing | 0.780 | 0.860 | 0.789-0.930 | 0.805 | 0.763 | 0.702 | 0.849 |

DL, deep learning; AUC, area under the curve; CI, confidence interval; PPV, positive predictive value; NPV, negative predictive value.

**Supplementary Figure 8.** ROC curves for different Combined DL models in training **(A),** validation **(B)** and testing **(C)** sets. ROC, receiver operating characteristic; DL, deep learning.

# 9 Delong Tests for Different Models

**Supplementary Figure 9.** Delong tests for different models in training **(A)**, validation **(B)** and testing **(C)** sets.

# 10 Calibration Curve Analysis

**Supplementary Figure 10.** Calibration curves for different models in training **(A)**, validation **(B)** and testing **(C)** sets.

# 11 DCA Curve Analysis

**Supplementary Figure 11.**  DCA curves for different models in training **(A)**, validation **(B)** and testing **(C)** sets. DCA, decision curve analysis.
